# Increased rate of enteric bacteria as cause of periprosthetic joint infections in patients with liver cirrhosis

**DOI:** 10.1186/s12879-022-07379-2

**Published:** 2022-04-19

**Authors:** Uta S. Koepf, Sebastian Scheidt, Gunnar T. R. Hischebeth, Christian P. Strassburg, Dieter C. Wirtz, Thomas M. Randau, Philipp Lutz

**Affiliations:** 1 Department of Internal Medicine I, University Hospital Bonn, Venusberg Campus 1, 53127 Bonn, Germany; 2 Department of Orthopaedics and Traumatology, University Hospital Bonn, Bonn, Germany; 3 Institute for Medical Microbiology, Immunology and Parasitology, University Hospital Bonn, Bonn, Germany

**Keywords:** PJI, Rifampicin, Rifaximin, Enteric bacteria, Liver cirrhosis, TKA, THA

## Abstract

**Introduction:**

Periprosthetic joint infections (PJI) are a major complication in joint-arthroplasty. Rifampicin is often used as an additional agent to treat PJI, because it penetrates bacterial biofilms. However, rifaximin, belonging to the same antibiotic class as rifampicin, is frequently used to prevent episodes of hepatic encephalopathy in patients with cirrhosis and may induce resistance to rifampicin. The aim of this study was to examine the microbial pattern of periprosthetic joint infections in cirrhotic patients and to test the hypothesis that intake of rifaximin increases the rate of resistance to rifampicin in periprosthetic joint infections.

**Methods:**

A cohort of cirrhotic patients and PJI (n = 25) was analysed on the characteristics of bacterial isolates from sonication and tissue analysis. In a second step a subgroup analysis on the development of rifampicin resistant bacterial specimens, depending on the intake of rifaximin (8 rifaximin intake patients vs. 13 non rifaximin intake patients) was performed.

**Results:**

Intestinal bacteria were found in 50% of the specimens, which was significantly more frequent than in a control cohort. By comparison of the single bacterial isolates, rifampicin resistance was detected in 69.2% (9/13) of the rifaximin-intake samples. In contrast, the non-rifaximin-intake isolates only were resistant to rifampicin in 22.2% (4/18) of the cases (p = 0.01). The odds ratio for developing a rifampicin-resistance through rifaximin intake was calculated as OR = 13.5.

**Conclusion:**

Periprosthetic joint infections have a high incidence of being caused by enteric bacteria in cirrhotic patients. Due to this change in microbial pattern and the innate resistance to rifampicin of most of gram-negative bacteria, the therapy with rifampicin should be carefully considered. The association between the use of rifaximin and developed resistance to rifampicin has a major impact on the treatment of PJI.

## Introduction

The risk for periprosthetic infections, estimated at around 1–2% for total knee arthroplasties (TKA) and 1% for total hip arthroplasties (THA) in the general population [1, 2] increases to 3.7% for THA and around 2.7% for TKA in cirrhotic patients [3]. Due to a compromised antibacterial immune response in cirrhotic patients [4, 5], periprosthetic joint infections (PJI) become even more a disastrous and feared complication [6]. Gut microbiome alterations (dysbiosis) in cirrhotic patients are frequently reported [7–11] and lead to higher abundance and relative overgrowth of *Staphylococcaeae, Enterobacteriaceae* and *Enterococcaceae* [7, 11]*.* A Europe-wide study showed that, due to bacterial translocation from the intestine, bacterial infections in cirrhotic patients are mainly caused by gram-negative bacteria such as *Escherichia coli, Klebsiella pneumonia* [12].

In non-cirrhotic patients, common pathogens in periprosthetic infections are biofilm-forming species such as *Staphylococcus* species [13, 14]. The foreign body of the prosthesis provides a surface where bacterial exopolysaccharides can adhere [15, 16], increasing the minimum inhibitory concentration (MIC) up to 100–1000 times [15], which leads to low susceptibility to antibiotic treatment [16]. Therapy in those cases requires a prolonged antibiotic treatment, preferably with a drug combination that is effective against biofilm bacteria, including rifampicin (around 70% [17]) or doxycycline [18].

With its potent activity against a variety of pathogens and potential to penetrate biofilms, rifampicin, which inhibits the bacterial RNA synthesis by binding and blocking the beta subunit of the DNA-dependent-RNA-polymerase [19], is a widely used antibiotic drug in joint infections [20]. Rifampicin-resistant pathogens are also known to be cross-resistant to other approved rifamycins (rifambutin, rifaximin and rifapentine) [21]. Rifaximin, which is characterized by poor intestinal absorption, prevents episodes of hepatic encephalopathy (HE) in patients with cirrhosis [22–27], so that consensus guidelines recommend long-term rifaximin use along with non-absorbable disaccharides in patients with recurrent episodes of HE [28].

Resistance to rifamycin in *Staphylococcus aureus* is mediated primarily by mutations in the rpoB gene [29], but seems to be reversible after months without rifampicin [21]. In patients with cirrhosis, long-term intake of rifaximin, despite the low plasma concentration, was associated with appearance of rifampicin-resistance in skin colonizing *Staphylococcus* species [27, 30, 31]. Three months after the end of treatment, the mutant population is once again overcome by the wildtype strain [30, 32].

The aim of this study was to examine the microbial pattern of periprosthetic joint infections in cirrhotic patients and to test the hypothesis that intake of rifaximin increases the rate of resistance to rifampicin in periprosthetic joint infections.

## Methods

For this retrospective cohort study a database search was performed for patients with the combination of liver cirrhosis and periprosthetic joint infections, who were admitted between January 2009 and September 2020 at the University Hospital of Bonn. Patients were excluded if surgical or antibiotic treatment was started prior to admission, or if no bacteria was isolated from intraoperative specimens. Furthermore, no fungi or mycobacteriacae were considered.

Initially, the database search retrieved 45 patients, of whom 20 patients had to be excluded, because diagnosis of PJI of hip or knee was not confirmed or essential data were missing (14 patients), PJI was caused by *Candida* species in four patients, in one patients no microorganism could be detected and one patient was affected by *Mycobacterium tuberculosis*. Finally, 25 patients with PJI with 60 bacterial strains were included in the analysis (Tables 1 and 2). The leading cause for cirrhosis in the study cohort was alcohol, followed by viral hepatitis, while none of the patients suffered from an autoimmune or biliary cause. Cirrhosis classification scores were calculated upon operation date.

**Table 1:** Patients characteristics

	All (n = 25)	RI-group (n = 8)	NRI-group (n = 11)
Patients demography			
Female	*13*	*3*	*7*
Male	*12*	*5*	*4*
Age (years)^*#^	*60.3 (30–77;* ± *11)*	*60.5 (38–75;* ± *11.9)*	*62.8 (50–77;* ± *7)*
Characteristics of joints and rifaximin intake			
THA	*18*	*5*	*9*
TKA	*7*	*3*	*2*
Rifaximin intake	*8*	*8*	*/*
Duration of rifaximin intake (months)^*#^	*/*	*24.75 (4–60;* ± *18)*	*/*
Characteristics of liver cirrhosis in patients			
Cirrhosis underlying disease			
Alcoholic cirrhosis	*14*	*4*	*8*
Viral hepatitis	*5*	*2*	*2*
Drug toxicity	*1*	*1*	*/*
Post-ischemic/mof	*1*	*/*	*1*
Unknown origin	*2*	*1*	*1*
NAFLD	*2*	*/*	*1*
MELD^*#^	*13.4 (6–36;* ± *6.6)*	*11.9 (8–18;* ± *3)*	*16.6 (10–36;* ± *8.1)*
Child Pugh			
A	*16*	*4*	*8*
B	*7*	*4*	*1*
C	*2*	*/*	*2*
Survival after 6 months	*14/25*	*5/8*	6/11

Characteristics of the patients and the underlying diseases, *TKA* total knee arthroplasty, *THA* total hip arthroplasty, *MELD* Model of end stage liver disease score, *NAFLD* non-alcoholic fatty liver disease, *mof* multiorgan failure, *RI-group* patients with rifaximin intake, *NRI-group* patients without rifaximin intake

^*^Standard deviation; #Minimum/maximum

**Table 2:** Characteristics of bacterial isolates

Bacterial isolates	Total (n = 60)		RI-group (n = 13)		NRI-group (n = 18)	
	n	%	n	%	n	%
*Staphylococcus aureus*	5	8.3	1 (0)	7.7	4 (0)	22.2
*Staphylococcus epidermidis*	12	20	5 (5)	38.5	7 (1)	38.9
*Staphylococcus haemolyticus*	3	5	2 (2)	15.4	1 (0)	5.6
*Staphylococcus hominis*	3	5	2 (0)	15.4	1 (0)	5.6
*Staphylococcus lugdunensis*	1	1.7	1 (0)	7.7	/	/
*Staphylococcus intermedius*	1	1.7	/	/	1 (0)	5.6
*Streptococcus mitis/oralis*	1	1.7	/	/	/	/
*Streptococcus salivarius*	1	1.7	/	/	/	/
*Cutibacterium acnes*	2	3.3	1 (1)	7.7	1 (0)	5.6
*Enterococcus faecalis*	7	11.7	1 (1)	7.7	1 (1)	5.6
*Enterococcus faecium*	6	10	/	/	1 (1)	5.6
*Enterococcus hirae*	1	1.7	/	/	1 (1)	5.6
*Clostridioides difficile*	1	1.7	/	/	/	/
*Enterobacter cloacae*	4	6.7	/	/	/	/
*Pseudomonas aeruginosa*	4	6.7	/	/	/	/
*Escherichia coli*	2	3.3	/	/	/	/
*Klebsiella oxytoca*	1	1.7	/	/	/	/
*Proteus mirabilis*	2	3.3	/	/	/	/
*Proteus vulgaris*	1	1.7	/	/	/	/
*Serratia marcescens*	2	3.3	/	/	/	/

(n) amount of isolates with resistance to Rifampicin

Bacterial isolates in the NRI-/RI-group (Rifaximin intake or no Rifaximin intake) and susceptibility to Rifampicin in both groups (in brackets)

In the first step of the analysis the cohort was examined for the overall microbial pattern of the periprosthetic joint infections. To compare the pattern of bacteria in our patients with cirrhosis to a cohort of general patients with PJI, we used data from a previous study on microbiological diagnostic methods of PJI from our university [33]. In the second step of the analysis, all bacterial isolates with unknown susceptibility or with innate resistance to rifampicin were excluded and patients then assigned either to the rifaximin-intake or the no-rifaximin group. Hence 8 patients were assigned to the rifaximin-intake and 13 to the no-rifaximin intake group.

Bacteria were identified through bacteriological cultures of tissue and sonication [34]. Additional statistical analysis was performed using IBM SPSS version 22 (SPSS Inc, IBM, Chicago, IL) for patient age, sex, duration of rifaximin-intake, the MELD-Score (Model for End-Stage Liver Disease) and the Child–Pugh-Score. Normality was assessed by using histograms and equality of variances by using the Shapiro–Wilk test. Demographic characteristics and read-outs of different findings as well as quantitative parameters were compared by using the Mann–Whitney-U test. For comparison of qualitative parameters, Fisher exact test was used. To classify the risk to develop a resistance to rifampicin when taking rifaximin Odds ratio was computed. Continuous data are reported as mean (standard deviation, SD) or median (minimum–maximum, MIN/MAX). The reported p values are 2-sided, with a significance level of 0.05. A post-hoc power analysis was performed with G-Power (University of Dusseldorf, Germany). The study was approved by the local ethic committee (330/19) and conducted according to the principles of the declaration of Helsinki.

## Results

Patients’ demography revealed a balanced distribution of age and sex. The joint infections affected total hip arthroplasties (18/25; 72%), knee arthroplasties (7/25; 28%). The underlying diseases of cirrhosis were in 56% alcohol abuse (14/25) and viral hepatitis (5/25; 20%).

In more than half of the samples gram-positive bacteria were detected (44/60; 73.3%), with Staphylococci and Streptococci being the biggest fraction (27/60; 45%) (Table 2). In 26.7% (16/60) of the specimen gram-negative bacteria such as *Escherichia coli, Enterobacter cloacae, Klebsiella oxytoca, Proteus* species*, Serratia marcescens* and *Pseudomonas aeruginosa* were found. 37 of all strains found are commonly known for the capability of producing biofilms (61.7%). Most of the 25 (20/25, 80%) Staphylococcal *strains (S. epidermidis, S. haemolyticus, S. intermedius* and *S. lugdunensis)* were coagulase-negative and 20% were *S. aureus (5/25).* Table 3 displays the susceptibility to the most important substance groups of antibiotics. *S. epidermidis* was mostly resistant to ampicillin/sulbactam (11/12; 91.7%). Interestingly, when we compared the occurrence of intestinal bacteria as cause of PJI to a control cohort, we found that enteric bacteria were significantly more frequent in PJI from cirrhosis patients while staphylococci were less frequent (Table 4).

**Table 3:** Resistogram of bacterial isolates

Bacterial isolates	Total (n = 60)	Ampicillin Sulbactam			Piperacilline Tazobactam			Cephalosporin^a^			Carbapeneme^b^			Fluorquinolone^c^		
	n	r	s	n	r	s	n	r	s	n	r	s	n	r	s	n
*Staphylococcus aureus*	5	1	3	1	1	3	1	1	3	1	1	3	1	2	2	1
*Staphylococcus epidermidis*	12	11	0	1	8	0	4	10	0	2	4	0	8	9	2	1
*Staphylococcus haemolyticus*	3	3	0	0	3	0	0	1	0	2	0	0	3	3	0	0
*Staphylococcus hominis*	3	1	2	0	1	2	0	1	2	0	0	0	3	1	2	0
*Staphylococcus lugdunensis*	1	0	1	0	0	1	0	0	1	0	0	1	0	0	1	0
*Staphylococcus intermedius*	1	1	0	0	0	0	1	0	0	1	0	1	0	0	1	0
*Streptococcus mitis/oralis*	1	0	1	0	0	1	0	0	1	0	0	0	1	0	1	0
*Streptococcus salivarius*	1	0	1	0	0	1	0	0	1	0	0	0	1	0	1	0
*Cutibacterium acnes*	2	0	2	0	0	1	1	0	0	2	0	2	0	0	1	1
*Enterococcus faecalis*	7	0	6	1	0	6	1	6	0	1	0	6	1	1	3	3
*Enterococcus faecium*	6	6	0	0	6	0	0	6	0	0	6	0	0	3	0	3
*Enterococcus hirae*	1	1	0	0	1	0	0	1	0	0	1	0	0	1	0	0
*Clostridioides difficile*	1	0	0	1	0	0	1	0	0	1	0	0	1	0	0	1
*Enterobacter cloacae*	4	4	0	0	0	4	0	1	3	0	0	4	0	0	4	0
*Pseudomonas aeruginosa*	4	2	0	2	2	2	0	3	0	1	0	4	0	0	4	0
*Escherichia coli*	2	1	1	0	0	2	0	0	2	0	0	2	0	2	0	0
*Klebsiella oxytoca*	1	0	1	0	0	1	0	0	1	0	0	1	0	1	0	0
*Proteus mirabilis*	2	1	1	0	0	2	0	0	2	0	0	2	0	0	2	0
*Proteus vulgaris*	1	1	0	0	0	1	0	0	1	0	0	1	0	0	1	0
*Serratia marcescens*	2	2	0	0	0	2	0	1	0	1	2	0	0	2	0	0

Resistogram of all bacterial isolates of the most important substance groups of antibiotics

r = resistant, s = sensitive, n = not indicated

^a^Ceftriaxon/Cefuroxim, ^b^Meropenem/Imipenem, ^c^Ciprofloxacin/Levofloxacin

**Table 4:** Bacterial pathogens in the cirrhosis and a control cohort

Bacterial isolates	Cirrhosis (n = 60)		Control cohort (n = 43)		p
	n	%	n	%	
Enterococci *and Enterocateriaceae*	30	50	7	16	**0.0004**
Staphylococci	25	42	28	65	**0.02**
Streptococci	2	3	3	7	0.65

Bacterial pathogens from the major three groups in the cirrhosis cohort compared to control cohort from our university published previously [33]

**Fig. 1:**
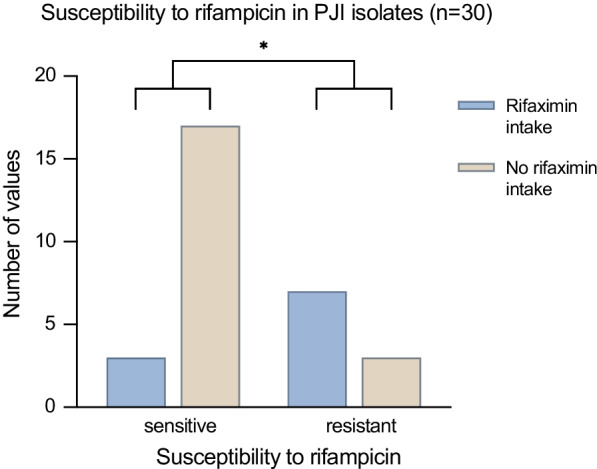
In periprosthetic joint infection, both groups (RI; NRI) had sensitive and resistant microbes, but differ in regard to their rifaximin intake; *significant difference (p = 0.01)

By comparison of the single bacterial isolates, rifampicin resistance could be detected in 69.2% (9/13) of the microbiological cultures from patients of the rifaximin-intake group. By contrast, the non-rifaximin-intake isolates only were resistant to rifampicin in 22.2% (4/18) of the cases (p = 0.01, see Fig. 1). The odds ratio for developing rifampicin-resistance by taking rifaximin was calculated as 13.5. Post-hoc power analysis revealed a medium to high power (0.73) and a high effect (d = 0.86).

No association between susceptibility to rifampicin and age (p = 0.6), MELD-Score (p = 0.92) or sex (p = 0.35) could be revealed. No significant correlation was found between susceptibility to rifampicin and the individual duration of rifaximin intake (p = 0.2). Within 6 months post-operatively 11 of 25 (44%) patients in this cohort died. Survival after six months was not dependent on the susceptibility to rifampicin (p = 0.66).

## Discussion

Out data indicate that PJIs in cirrhotic patients are in 26.7% (16/60) of the cases associated with gram-negative bacteria, while non-cirrhotic patients mostly suffer from PJIs caused by CoNS (30–43%), *Staphylococcus aureus* (12–23%) or Streptococci (9–10%) [35–39]. Gram-negative bacteria (3–6%) or Enterococci (3–7%) are found less often in non-cirrhotic patients, which highlights the importance of our findings [17, 39, 44]. When comparing the occurrence of intestinal bacteria in our cohort to a control cohort from our university, we found a significantly higher rate of intestinal pathogens in PJI from cirrhosis patients. Our data indicate for the first time that periprosthetic joint infections in patients with cirrhosis are often caused by intestinal pathogens, strengthening the concept that bacterial translocation from the intestine and alterations in the microbiome play a major role for infections in those patients [40]*.* The difference in the microbial pattern in this cohort coincides with the findings of bacterial dysbiosis and other bacterial infections in cirrhotic patients [12]*.* Gut microbiome transition due to cirrhosis and alcohol seems to induce differences in bacterial colonisation all over the human body system. Concordantly to that, most of the examined patients suffered from alcoholic cirrhosis (56%), where microbiome transition is described most elaborately [7, 10, 41]. The underlying reasons for gut microbiome transition are yet fully understood. Therapeutic considerations should include the higher rate of intestinal bacteria with a larger amount of gram-negative and anaerobic bacteria and hence a shift in susceptibility to antibiotic agents.

The detected difference between the RI- and the NRI-group suggests that rifaximin may induce rifampicin resistance in bacteria causing PJIs. In this cohort the resistance did not seem to be dependent on the duration of rifaximin intake, which might be due to the fact that all patients had been taking rifaximin for at least 4 weeks prior to development of PJI and because resistance to rifaximin can be detected early [42]. It has earlier been reported that intake of rifaximin may induce cross-resistance to rifampicin [30, 31] in healthy individuals. However, this is the first study to analyse the impact of rifaximin intake on the microorganisms causing periprosthetic joint infections in cirrhotic patients.

In an in-vitro study Rothstein et al. described that cross-resistances among rifamycin derivates have a great impact on the therapeutical benefit of these antibiotics. The reported resistance regularly occurred during intake, but rapidly disappeared after discontinuation of the drug [21]. In almost 50% of 198 skin bacterial isolates, especially *Staphylococcus* species, from 25 patients, Chang et al. found resistance to rifampicin during the intake of rifaximin [30]. In accordance to the results from Rothstein et al. the prevalence of resistance decreased after stopping rifaximin therapy [30]. In contrast to that, Valentin et al. showed remaining rifampicin-resistant strains nine weeks after discontinuation of rifaximin [31]. However, rifaximin therapy is usually given for long periods of time in patients with cirrhosis. Though, the administration of rifampicin in cirrhotic patients is never uncomplicated due to liver-related side effects.

The cohort of this study suffered from a relatively high mortality rate of 40% (10/25). In the literature, cirrhotic patients are nearly ten times more likely to die after joint infections as patients without liver disease [3]. In this cohort, we did not detect any hints for a significantly higher mortality rate in the subgroup of patients with rifampicin resistance. Our cohort, however, suffered in 31.7% from obligate and facultative anaerobic bacterial infections (*Clostridium difficile, Cutibacterium acnes, Enterobacter cloacae, Escherichia coli, Serratia marcescens, Klebsiella oxytoca, Proteus* species*, Pseudomonas aeruginosa*; 19/60), although the literature describes a portion of only 3–6% in PJI [43].

Even if the cohort of this study is small, our findings indicate that the patients’ medical history with regard to former or current rifaximin intake should be carefully noted. As rifampicin is widely used due to its singular bactericidal activity within biofilm, alternative antibiotics for patients with rifampicin resistance are scarce. As most cirrhotic patients on rifaximin suffer from multimorbidity, such as peripheral arterial disease, osteoporosis, cardiovascular disease, they have an elevated prevalence of joint implants, which may become infected due to the compromised immune system. Some gram-negative bacterial strains found in this study are intrinsically resistant to rifampicin. Because rifampicin is a widely used antibiotic in periprosthetic joint infection, this shift has to be seriously considered in the empirical antibiotic treatment of cirrhotic patients.

Our study is limited by the small sample size. However, even in a big tertiary centre, joint replacement in the small, but important subgroup of patients with liver cirrhosis is not frequent, and PJI is even more rare. Nevertheless, due to its severe consequences for the individual patient, these infections require particular attention. The results of our study indicate that it would be of high interest to investigate the microbial pattern of PJI and the influence of rifaximin in patients with cirrhosis on a bigger scale. As in all microbiological studies, our results from a European centre may not be applicable to other areas of the world.

## Conclusion

Periprosthetic joint infections might be caused more often by enteric bacteria in patients with liver cirrhosis than expected from patients without cirrhosis. Due to this change in microbial pattern and the innate resistance to rifampicin of most of gram-negative bacteria, the therapy with rifampicin should be carefully considered. Additionally, the association between the use of rifaximin and developed resistance to rifampicin has a major impact on the treatment of periprosthetic joint infections in this cohort. Before empiric antibiotic therapy is started, careful attention should be paid to the medical history in patients with liver cirrhosis.

## Data Availability

All data generated or analysed during this study are included in this published article.

## Abbreviations

MIC: Minimum inhibitory concentration
MELD: Model of End Stage Liver Disease-Score
NAFLD: Nonalcoholic fatty liver disease
RI-group: Rifaximin intake group
NRI-group: No Rifaximin intake group
PJI: Periprosthetic joint infections
TKA: Total knee arthroplasty
THA: Total hip arthroplasty
HE: Hepatic encephalopathy
CoNS: Coagulase negative Staphyloccoci
